# Face expression recognition based on NGO-BILSTM model

**DOI:** 10.3389/fnbot.2023.1155038

**Published:** 2023-03-21

**Authors:** Jiarui Zhong, Tangxian Chen, Liuhan Yi

**Affiliations:** College of Electrical Engineering and New Energy, China Three Gorges University, Yichang, China

**Keywords:** northern goshawk algorithm, NGO-BILSTM model, face recognition, facial expression, hyperparameter optimization

## Abstract

**Introduction:**

Facial expression recognition has always been a hot topic in computer vision and artificial intelligence. In recent years, deep learning models have achieved good results in accurately recognizing facial expressions. BILSTM network is such a model. However, the BILSTM network's performance depends largely on its hyperparameters, which is a challenge for optimization.

**Methods:**

In this paper, a Northern Goshawk optimization (NGO) algorithm is proposed to optimize the hyperparameters of BILSTM network for facial expression recognition. The proposed methods were evaluated and compared with other methods on the FER2013, FERplus and RAF-DB datasets, taking into account factors such as cultural background, race and gender.

**Results:**

The results show that the recognition accuracy of the model on FER2013 and FERPlus data sets is much higher than that of the traditional VGG16 network. The recognition accuracy is 89.72% on the RAF-DB dataset, which is 5.45, 9.63, 7.36, and 3.18% higher than that of the proposed facial expression recognition algorithms DLP-CNN, gACNN, pACNN, and LDL-ALSG in recent 2 years, respectively.

**Discussion:**

In conclusion, NGO algorithm effectively optimized the hyperparameters of BILSTM network, improved the performance of facial expression recognition, and provided a new method for the hyperparameter optimization of BILSTM network for facial expression recognition.

## 1. Introduction

The change of facial expression can reflect the change of human emotions and psychology, which plays an indispensable role in daily life (Li and Deng, 2020; Revina and Emmanuel, 2021). Human beings express their emotions mainly through language, voice tone, body movements and facial expressions, and facial expressions contain a large amount of effective information, which can convey the real emotions of human hearts and are more accurate than the information conveyed by language expressions (Li et al., 2018; Minaee et al., 2021; Yang et al., 2021).

In recent years, a variety of AI devices have come into the public eye, and Artificial intelligence algorithms are developing rapidly (Prajapati et al., 2021; Ramachandran and Rajagopal, 2022; Ravinder et al.). The public hopes that computers can understand and express their own emotions through facial expression recognition like humans do, and also give correct feedback according to users' emotional needs (Li et al., 2020; Zhang, 2020).

Exploring face expression recognition technology can provide technical support for artificial intelligence emotional expression. The literature (Han et al., 2022) proposes a new HRL model, which uses the universal matching measure to dynamically display the discriminant learning constraint features in facial expression recognition, and develops an example demonstration. The literature (Gao et al., 2020) proposes a face recognition method based on plural data enhancement, which uses the information provided by the original face image for feature extraction and then fuses the original image with the new feature image to obtain a synthetic plural image that can perform face image recognition under non-ideal conditions. The literature (Gurukumar et al., 2021) plans the facial expression recognition model with the help of artificial intelligence techniques, which mainly includes the steps of data acquisition, face detection, optimal feature extraction and emotion recognition, and uses the optimal scale-invariant feature transform for face expression feature extraction and hybrid metaheuristic algorithm for optimizing the key points that give unique information, which in turn performs facial expression recognition.

In addition, in literature (Zhao et al., 2020), a new deep neural network is constructed to deeply encode the face region and a new face alignment algorithm is proposed. The Literature (Lu, 2021) proposes a multi-angle facial expression recognition method, which is based on generative adversarial network for feature mapping and CNN for classification and learning. The literature (Cao et al., 2021) proposed a method of facial expression recognition by Fourier frequency transform, and obtained the correct facial expression information by adjusting the frequency band of the wrong expression. The Literature (Liao and Gu, 2020) proposes a face recognition method based on subspace extended sparse, which uses subspace extended sparse representation classifier for recognition.

In the latest research on facial expression recognition, the Literature (Li et al., 2017) propose a new DLP-CNN (Deep Locality-Preserving CNN) method, which aims to enhance the discriminative power of deep features by preserving the locality closeness while maximizing the inter-class scatters. In the Literature (Li et al., 2018), a convolutional neural network (CNN) with an attentional mechanism (ACNN) is proposed for facial expression recognition in the field, which can perceive the obscured area of the face and focus on the most discriminating unobscured area. Two versions of ACNN have been introduced: the patch-based ACNN (pACNN) and the global-local ACNN (gACNN). pACNN only focuses on partial facial patches. gACNN combines a local representation at the patch level with a global representation at the image level. The Literature (Chen et al., 2020) propose a novel approach named Label Distribution Learning on Auxiliary Label Space Graphs (LDL-ALSG) that leverages the topological information of the labels from related but more distinct tasks, such as action unit recognition and facial landmark detection. The underlying assumption is that facial images should have similar expression distributions to their neighbors in the label space of action unit recognition and facial landmark detection.

In order to explore the application of NGO-BILSTM model in facial expression recognition, this paper analyzes the debate in three parts. The first part introduces the basic principles of NGO algorithm. In the second part, LSTM neural network is introduced, pointing out that LSTM can not obtain reverse information, and a BILSTM neural network combining forward LSTM and reverse LSTM is introduced. The network can form two independent networks with opposite data flows, and can simultaneously process data with positive and negative flows. Then, according to the NGO algorithm, the hyperparameters of BILSTM were optimized to build the NGO-BILSTM model for facial expression recognition. The third part is the result analysis. According to the constructed NGO-BILSTM facial expression recognition model and the accuracy evaluation index of confusion matrix, three facial expression data sets of FER2013, FERPlus and RAF-DB are provided to evaluate the performance of this model. The validity and feasibility of the proposed model in face expression recognition are measured by the accuracy.

## 2. NGO-BILSTM model

Based on the NGO algorithm and BILSTM neural network, combining the advantages of the two algorithms, this paper builds a NGO-BILSTM model to provide a better technical basis for artificial intelligence emotional expression.

### 2.1. NGO algorithm

#### 2.1.1. Initializing the NGO algorithm

In the NGO algorithm, the population number and location of the northern goshawk can be represented by the following population matrix, namely:


$$\begin{array}{l} X={\left[\begin{array}{c} X_{1}\\ \vdots \\ X_{i}\\ \vdots \\ X_{\text{N}} \end{array}\right]}_{m\times N}={\left[\begin{array}{ccccc} x_{1,1} & \cdots & x_{1,j} & \cdots & x_{1,m}\\ \vdots & \ddots & \vdots & \vdots \\ x_{i,1} & \cdots & x_{i,j} & \cdots & x_{i,m}\\ \vdots & \vdots & \ddots & \vdots \\ x_{N,1} & \cdots & x_{N,j} & \cdots & x_{N,m} \end{array}\right]}_{m\times N} \end{array}$$

In the NGO algorithm, the objective function value of the northern eagle population is represented by a vector, i.e.:


$$\begin{array}{l} F={\left[\begin{array}{c} F_{1}\\ \vdots \\ F_{i}\\ \vdots \\ F_{\text{N}} \end{array}\right]}_{1\times N}={\left[\begin{array}{c} F\left(X_{1}\right)\\ \vdots \\ F\left(X_{i}\right)\\ \vdots \\ F\left(X_{N}\right) \end{array}\right]}_{1\times N} \end{array}$$

#### 2.1.2. NGO's prey identification

In the first stage, the prey selection and aggressive behavior of the northern goshawk is represented by the following mathematical formula:


(1) $$\begin{array}{l} P_{i}=X_{k} \end{array}$$

(2) $$\begin{array}{l} x_{i,j}^{new,P1}=\{\begin{array}{c} x_{i,j}+r\left(p_{i,j}-Ix_{i,j}\right),F_{p_{i}}<F_{i}\\ x_{i,j}+r\left(x_{i,j}-p_{i,j}\right),F_{p_{i}}\geq F_{i} \end{array} \end{array}$$

(3) $$\begin{array}{l} X_{i}=\{\begin{array}{c} X_{i}^{new,P1},F_{i}^{new,P1}<F_{i}\\ \;X_{i}\;,F_{i}^{new,P1}\geq F_{i} \end{array} \end{array}$$

Where *P*_*i*_ is the prey position, *F*_*p*_*i*__ is the objective function value of *P*_*i*_, *k* is a random integer in the range of [1, *N*]. $x_{i,j}^{new,P1}$ is the new position of the *j*th dimension of the *i*th northern goshawk, $F_{i}^{new,P1}$ is the objective function value of the *i*th northern goshawk based on the first stage update. *r* belongs to [0, 1], *I* is 1 or 2.

#### 2.1.3. NGO chase and escape

In the second stage, prey escape and the northern goshawk chasing prey are represented by the following mathematical formula:


(4) $$\begin{array}{l} X_{i,j}^{new,P2}=x_{i,j}+R\left(2r-1\right)x_{i,j} \end{array}$$

(5) $$\begin{array}{l} R=0.02\left(1-\frac{t}{T}\right) \end{array}$$

(6) $$\begin{array}{l} X_{i}=\{\begin{array}{c} X_{i}^{new,P2},F_{i}^{new,P2}<F_{i}\\ X_{i}\;,F_{i}^{new,P2}\geq F_{i} \end{array} \end{array}$$

Where *t* is the current iteration number, is the maximum iteration number (Dehghani et al., 2021).

### 2.2. BILSTM neural network algorithm

#### 2.2.1. LSTM neural network

**Figure 1:**
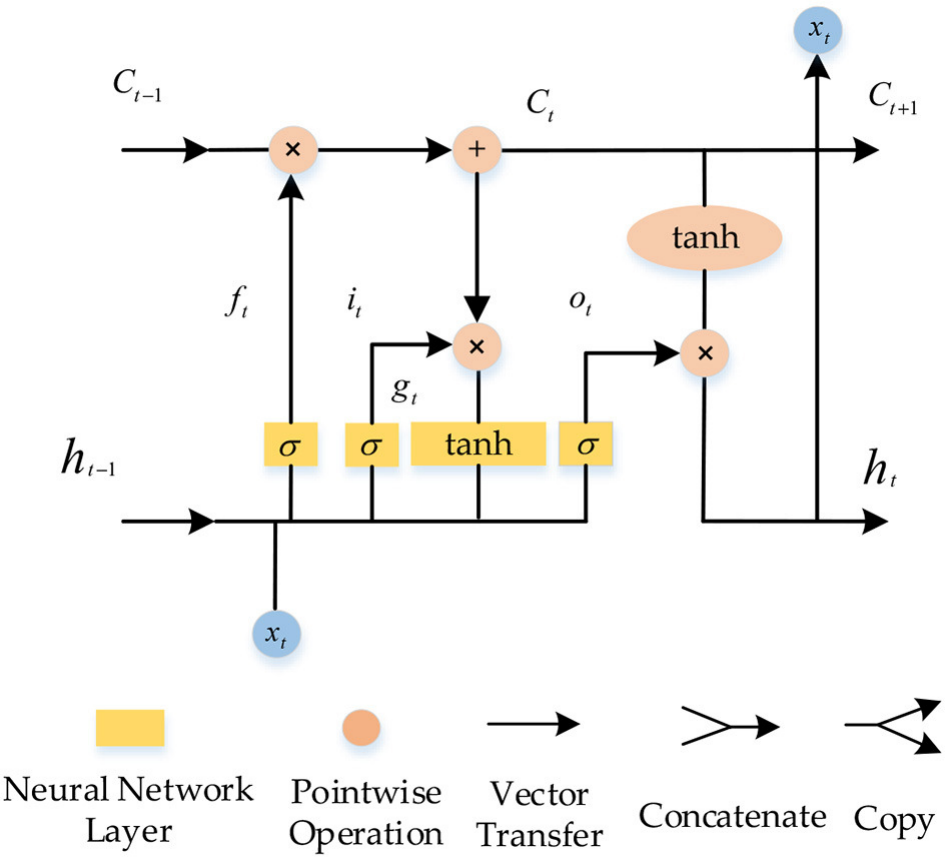
Neuronal structure of the LSTM.

LSTM is more efficient because the long-term memory network retains important in-formation for long-term memory and forgets other information to some extent, and sequential data processing is more efficient than recurrent neural networks. The neuron structure of LSTM is shown in Figure 1 (Bao et al., 2021; Zhou et al., 2022).

LSTM and RNN explore the dependencies between sequence elements through internal state transfer. However, LSTM introduces a gating mechanism to solve the short-comings of the RNN gradient update. The gating link of LSTM is divided into forgetting, input and output, and the state unit is introduced to regulate the operation of the whole network.

##### 2.2.1.1. Oblivious gating

*f*_*t*_ is the forgetting gate, which has the role of determining the degree of retention of the incoming information at the previous moment. The forgetting gate is obtained by linearly transforming the input at moment *t* with the output at moment *t* − 1 and then applying an activation function, which is calculated as:


(7) $$\begin{array}{l} f_{t}=\sigma \left(W_{f}x_{t}+U_{f}h_{t-1}+b_{f}\right) \end{array}$$

Where σ denotes the sigmoid activation function, *W*_*f*_ and *U*_*f*_ denote the weight matrix of the forgetting gate, *x*_*t*_ is the input, *h*_*t*−1_ is the implicit layer output, *b*_*f*_ denotes the bias value of the forgetting gate.

##### 2.2.1.2. Input gate

*i*_*t*_ is the input gate, it is mainly to decide the retention Chengdu of the information input at *t* moments. The input gate is calculated in a similar way to the forgetting gate, and its expression is:


(8) $$\begin{array}{l} i_{t}=\sigma \left(W_{i}x_{t}+U_{i}h_{t-1}+b_{i}\right) \end{array}$$

Where *W*_*i*_ and *U*_*i*_ denote the input gate weight matrix, *b*_*i*_ denotes the input gate bias value.

*g*_*t*_ is the input state, which is obtained from the implied layer output at moment *t* − 1 and the input at moment *t* by applying a tanh function through a linear transformation, whose expression is:


(9) $$\begin{array}{l} g_{t}=\tanh\left(W_{g}x_{t}+U_{g}h_{t-1}+b_{g}\right) \end{array}$$

Where *W*_*g*_ and *U*_*g*_ denote the temporary cell state weight matrix, denotes the temporary cell state bias value.

##### 2.2.1.3. State unit

The state unit of the LSTM is mainly used to update the internal state of the LSTM at the previous time to the internal state at this time. The formula calculates the internal state at this moment:


(10) $$\begin{array}{l} C_{t}=C_{t-1}\cdot f_{t}+g_{t}\cdot i_{t} \end{array}$$

##### 2.2.1.4. Output gate

*o*_*t*_ is the output gate, the output control of the output gate depends on the degree of the state unit, which is calculated as:


(11) $$\begin{array}{l} o_{t}=\sigma \left(W_{o}x_{t}+U_{o}h_{t-1}+b_{o}\right) \end{array}$$

Where *W*_*o*_ and *U*_*o*_ denote the weight matrix of the output gate, *b*_*o*_ denotes the bias value of the output gate.

The implied state output *h*_*t*_ at the final moment, which is determined by both the internal state and the output gate, is calculated as:


(12) $$\begin{array}{l} h_{t}=o_{t}\cdot \tanh\left(C_{t}\right) \end{array}$$

#### 2.2.2. BILSTM neural network

BILSTM neural network is proposed based on the LSTM network. BILSTM is composed of forward LSTM and reverse LSTM. The forward LSTM processes input data in the forward direction, while the reverse LSTM processes input data in the reverse direction. After processing, the output of the two LSTMS is joined together, namely, the output of BILSTM. BILSTM can transfer between past and future implied layer states and perform a feedback neural network, which can well uncover the implied connections between time series data. The BILSTM network can find the intrinsic links between the current moment data and the past and future data, which can improve the model testing accuracy and the data utilization efficiency.

**Figure 2:**
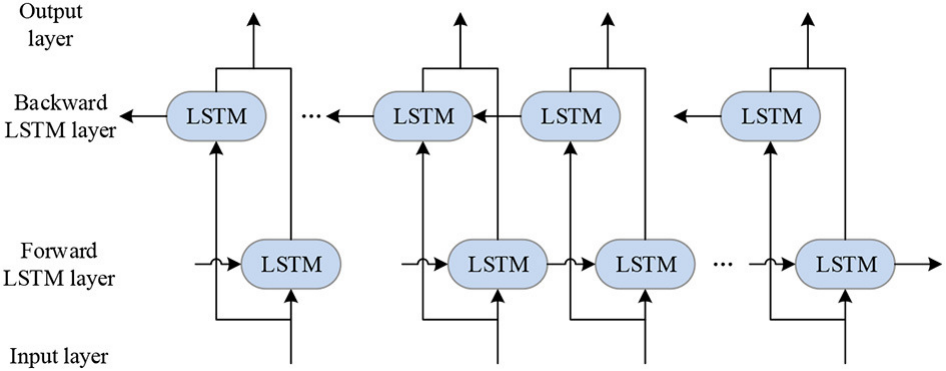
BILSTM structure diagram.

Structurally, compared with the one-way LSTM network, the BILSTM neural network is a two-way cyclic structure with forward and backward propagation. In terms of temporal structure, the flow of LSTM data is from the past to the future. In contrast, the flow of BILSTM data is added to the flow of data that will come to the past on top of the flow from the past to the future. The implied layers in the past and the implied layers in the future are independent of each other, so BILSTM can better explore the temporal characteristics of the data. BILSTM structure diagram is shown in Figure 2 (Gong et al., 2021; Hou and Zhu, 2021).

The above figure indicates no interaction between the positive and negative implicit layers, forming two independent networks with opposite data flow directions, which can handle models with both positive and negative flow directions.

The forward LSTM network computational expression is:


(13) $$\begin{array}{l} {\vec{h}}_{t}=LSTM\left(x_{t},{\vec{h}}_{t-1}\right) \end{array}$$

The inverse LSTM network computational expression is:


(14) $$\begin{array}{l} {\vec{h}}_{t}=LSTM\left(x_{t},{\vec{h}}_{t-1}\right) \end{array}$$

### 2.3. Face expression recognition model based on NGO-BILSTM

Based on the algorithmic advantages of the NGO algorithm and BILSTM neural network introduced in the previous section, this section combines the research characteristics of face recognition technology and constructs a facial expression recognition model based on NGO-BILSTM.

#### 2.3.1. Fairness in facial expression recognition

Facial expression recognition technology has become increasingly prevalent in recent years, with applications ranging from security and surveillance to emotion detection in marketing and healthcare. However, concerns about the fairness and accuracy of these systems have also emerged, particularly with respect to their impact on marginalized groups (Buolamwini and Gebru, 2018; Datta and Joshua Swamidass, 2021).

The issue of fairness in facial expression recognition technology is rooted in the fact that these systems are often trained on biased data sets, which can result in the perpetuation of existing societal biases. For example, if a data set used to train a facial recognition system is predominantly composed of images of white individuals, the system may perform poorly when trying to recognize the facial expressions of individuals with darker skin tones. This can result in inaccurate and unfair outcomes, such as misidentifying individuals of color as potential threats or suspects.

Another issue with facial expression recognition technology is that it may not be able to accurately detect or recognize expressions in individuals from certain cultures or backgrounds. For instance, some cultures may have different facial expressions for emotions such as happiness or sadness, and a facial recognition system trained on a data set with limited cultural diversity may struggle to accurately detect or recognize these expressions.

There are ongoing efforts to address the issue of fairness in facial expression recognition technology. One approach is to use more diverse and representative data sets to train these systems, in order to mitigate the impact of biases in the training data. Additionally, there have been calls for greater transparency and accountability in the development and deployment of these systems, including the use of third-party audits and evaluations to ensure that they are accurate and fair for all individuals, regardless of their race, ethnicity, gender, or other factors.

Overall, the issue of fairness in facial expression recognition technology is complex and multifaceted, and requires ongoing attention and effort to address. By working to ensure that these systems are accurate, transparent, and equitable for all individuals, we can help to mitigate the potential harms of biased and unfair technology, and create a more just and equitable society for all.

#### 2.3.2. Face expression data pre-processing

The BILSTM network is used to process the input image and crop out the face region, after which the face region is aligned to reduce the interference of noise. Since the training of the network requires a large amount of data, some datasets with a small number of images, such as the SFEW dataset, cannot be supported for training. Therefore, during the training process, data enhancement, such as random cropping, rotation angle, flipping, etc., is needed for the input data. The data enhancement techniques can effectively enrich the diversity of images in many datasets.

The images obtained after the preprocessing is completed are fed into the BILSTM network for feature extraction. During the training, NGO algorithms were used to optimize the hyperparameters. The input image is passed through the implicit layer for feature extraction and output feature map for feature recognition and classification, and finally, the expression category and recognition accuracy corresponding to the input image are output. The trained network model is loaded and tested using the test set in the network testing phase. The data can be enhanced in the testing phase to improve the robustness of the network model. For the facial expression recognition task, the recognition accuracy is usually used as a criterion to measure a good or bad network model, and the higher the recognition accuracy, the better the performance of the network model and the better the recognition classification.

As fairness is crucial in facial expression recognition, the selection of facial expression data sets should consider whether the data sets include factors such as race, gender, skin color and cultural background. In order to ensure the fairness of face recognition, three data sets FER2013, FERPlus and RAF-DB are selected in this paper. The three data sets are described below:

##### 2.3.2.1. FER2013 dataset

FER2013 is a dataset containing 35,887 images of facial expressions labeled with seven basic emotions (anger, disgust, fear, happiness, sadness, surprise, and neutral). The data set contains facial images of people from different countries and regions, so it covers people of different races, genders, ages and cultural backgrounds. The data distribution of this dataset is shown in Table 1.

**Table 1:** Distribution of expression categories in the FER2013 dataset.

**Expression category**	**Happy**	**Sadness**	**Fear**	**Surprise**	**Disgust**	**Anger**	**Neutral**	**Total**
Training set	3,274	4,286	4,154	3,274	415	4,021	5,032	24,456
Public test set	405	610	532	405	62	489	631	3,134
Private test set	398	647	504	398	64	457	612	3,080

##### 2.3.2.2. FERPlus dataset

FERPlus is an expanded version of FER2013 that includes images from FER2013, but for each image, provides more accurate emotional labels, including “uncertain” labels. FERPlus's emotional tags were collected by collecting human tagger tags on Amazon Mechanical Turk and applying model-based methods to filter and clean up. The data distribution of the FERPlus dataset is shown in Table 2.

**Table 2:** Distribution of expression categories in the FERPlus dataset.

**Expression category**	**Happy**	**Sadness**	**Fear**	**Surprise**	**Anger**	**Disgust**	**Disdain**	**Neutrality**	**Total**
Training set	7,246	2,961	501	3,014	1,998	106	112	8,482	24,420
Public test set	854	326	60	405	279	26	18	1,198	3,166
Private test set	892	378	78	394	265	15	18	1,087	3,127

##### 2.3.2.3. RAF-DB dataset

The RAF-DB dataset contains about 30,000 images of various expressions downloaded from the Web. All the images in the RAFDB dataset differ between subjects in multiple aspects, such as masking of the face, lighting conditions, age, head posture, ethnic skin color, and racial gender. Each faces facial expression image was independently labeled by ~ 30 to 40 trained coders, so the RAF-DB dataset is rich in images not only in terms of number but also in terms of expression images in various states. The data distribution of the RAF-DB dataset is shown in Table 3.

**Table 3:** Distribution of expression categories in the RAF-DB dataset.

**Expresion category**	**Happy**	**Sadness**	**Fear**	**Suprise**	**Disgust**	**Anger**	**Neutral**	**Total**
Training set	4,768	1,991	283	1,288	721	708	2,520	12,279
Test set	1,188	482	78	732	163	165	183	2,991

#### 2.3.3. Optimization of BILSTM network parameters based on the NGO algorithm

The BILSTM network has more parameters, and the parameters that have a greater impact on the facial expression recognition results are the number of LSTM hidden layers, batch size, learning rate, number of iterations, and the parameter selection of Adam optimizer. Before training, the parameters of BILSTM that have a great influence are optimized by using the northern hawk optimization algorithm, and the optimal parameters of the BILSTM network are obtained, and the parameter settings after optimization are shown in Table 4.

**Table 4:** Parameter setting after optimization search.

**Parameters**	**Value**
LSTM implied layers	128
Batch size	4
Learning rate	0.0005
Number of iterations	300
Adam	β_1_ = 0.99, β_2_ = 0.999

#### 2.3.4. NGO-BILSTM model training process

**Figure 3:**
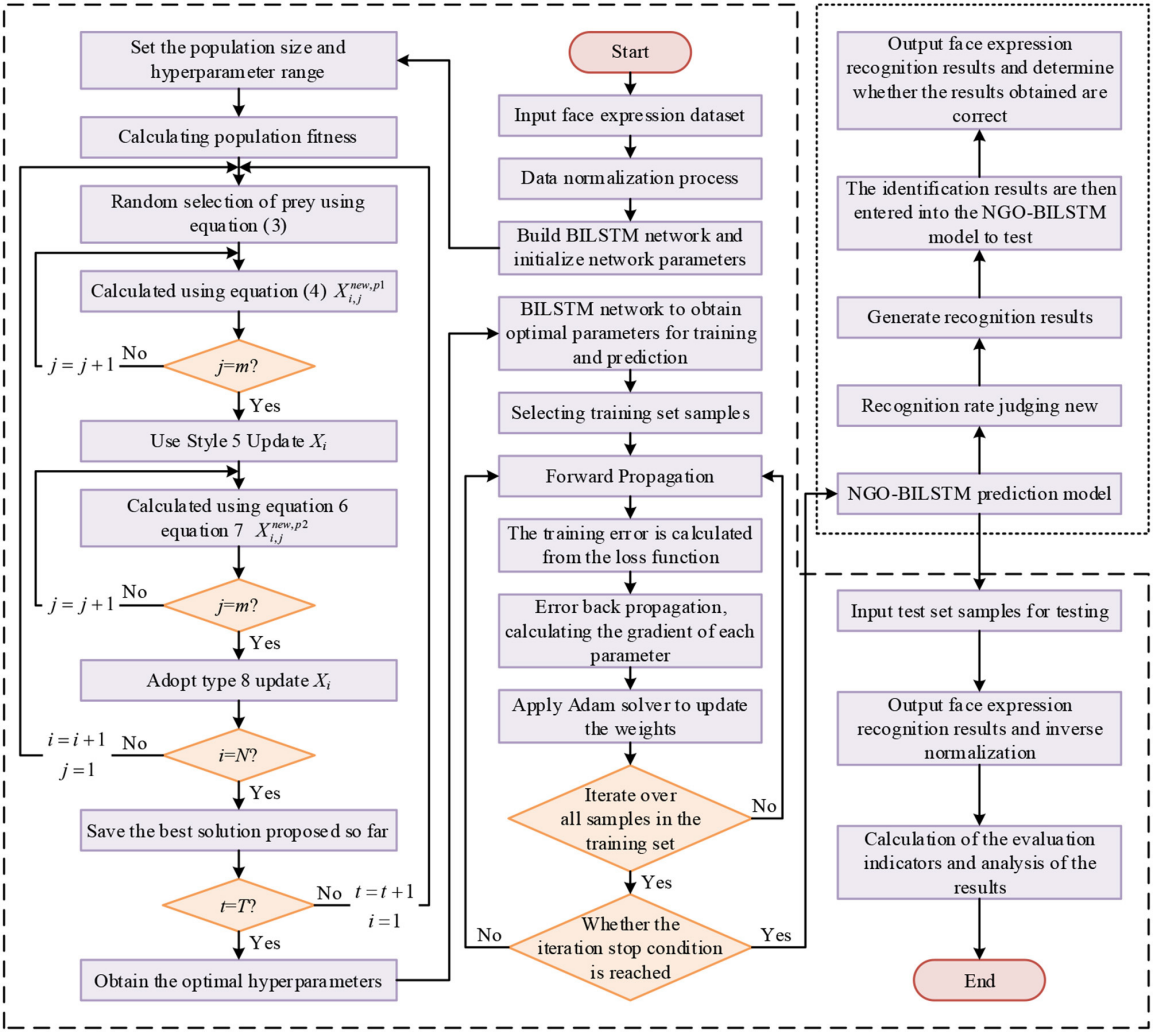
Flowchart of facial expression recognition based on NGO-BILSTM.

The NGO-BILSTM face expression recognition model is trained by pre-processing the face expression dataset and the BILSTM network after the optimization algorithm of the northern hawk, and the model training process uses the neural network backpropagation algorithm. The flow chart of the facial expression recognition model based on NGO-BILSTM is shown in Figure 3.

Firstly, the face expression dataset is divided into the training set and a test set, and the training set is normalized to the data. Secondly, the combined output values of forward and backward LSTM neurons are calculated according to the forward propagation algorithm. The error terms of the LSTM output layer are calculated according to the loss function, and then back propagated to the forward and backward LSTM implicit layers. The error terms of each LSTM neuron at the end of the implicit layer are calculated. Finally, the gradient of each weight is calculated based on the corresponding error term (Li et al., 2022), and the gradient descent-based optimization algorithm of the Adam optimizer is used to perform the weight update of the LSTM.

The Adam optimizer is an adaptive learning mechanism improved based on SGD architecture, which has the advantage of low dependence on the adaptive learning rate and the assignment of hyperparameters. The loss function is used to measure the difference between the predicted value and the real value. The smaller the value, the better the robustness of the model.

By setting the minimum value of the loss function as the optimization objective, the Adam optimizer updates the BILSTM weights continuously until the optimal face expression recognition model is obtained.

## 3. Analysis of facial expression recognition results based on the NGO-BILSTM model

Based on the previous design of the facial expression recognition process of NGO-BILSTM, this chapter mainly develops the experimental analysis to clarify the application of the NGO-BILSTM model in facial expression recognition.

### 3.1. Experimental preparation

#### 3.1.1. Experimental environment and data set

The experimental environment configuration of this chapter for facial expression recognition results is shown in Table 5.

**Table 5:** Experimental environment configuration.

**Environment configuration**	**Parameter configuration**
Operating systems	Windows 10 professional edition
GPU	Intel(R) Core(TM) i3-10100
Memory	8.00 GB
Accelerated libraries	CUDA 9.0
Programming languages	Python 3.8

In this chapter, face expression recognition experiments will be conducted on the FER2013 dataset, FERPlus dataset and RAF-DB dataset. The cross-entropy loss is used to optimize the network together with the Adam. The initial learning rate is set to 0.001, the momentum is set to 0.99, and 300 iterations are performed on both the FER2013 dataset, the FERPlus dataset and RAF-DB dataset, and the batch size is set to 15.

#### 3.1.2. Evaluation indicators of the NGO-BILSTM model

In the classification task of machine learning, we often use many metrics to measure the model's performance, such as ROC curve, PSI, recall, accuracy, F1 value, AUC value and confusion matrix. In this paper, we choose the confusion matrix to evaluate the performance of the NGO-BILSTM-based face expression recognition model.

The confusion matrix is the error matrix from which the recognition accuracy can be calculated. The confusion matrix in the classification task is used to reflect the probability that one of the total samples is predicted to be the remaining other samples, and its matrix size is generally *n* × *n*, *n* is the number of categories, and the confusion matrix is shown in Table 6.

**Table 6:** Confusion matrix.

**True value**	**Predicted value**	
	**Positive example**	**Negative example**
Positive example	TP	FN
Negative example	FP	TN

Accuracy is the ratio of the classification model's accurate prediction of a certain category of a given test set or the correct proportion of the whole sample predicted by the classification model, which is calculated by the formula:


(15) $$\begin{array}{l} Accuaracy=\frac{TP+TN}{TP+TN+FP+FN} \end{array}$$

### 3.2. Comparison and analysis of experimental results

To verify the effectiveness of the proposed model for facial expression recognition, this section tests and compares three types of face expression datasets, namely, the FER2013 dataset, FERPlus dataset and RAF-DB dataset, to validate the application of this paper's NGO-BILSTM model for facial expression recognition.

#### 3.2.1. FER2013 dataset

**Figure 4:**
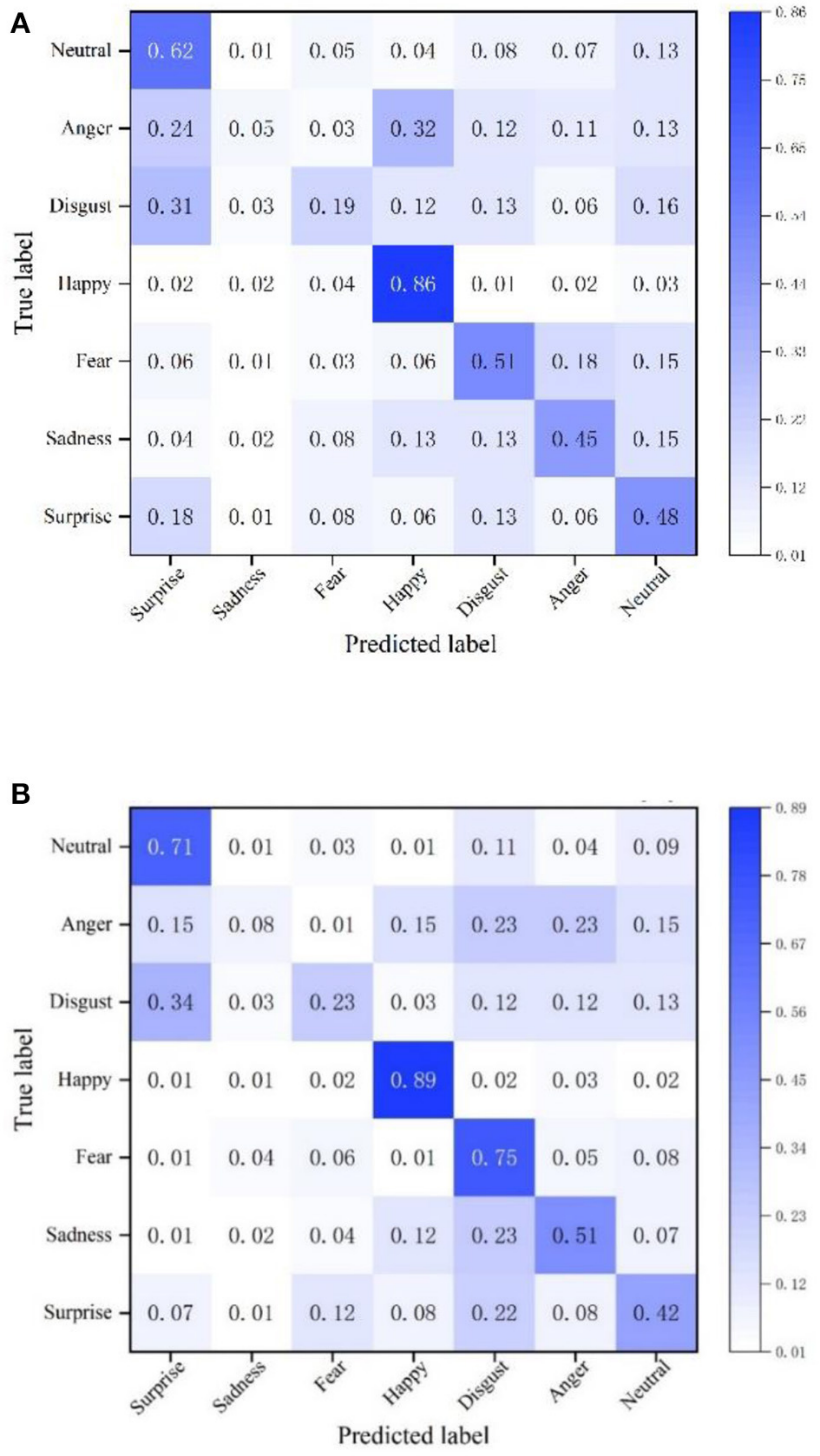
Confusion matrix on the FER2013 dataset. **(A)** Confusion matrix of VGG16 network. **(B)** The confusion matrix of the model in this paper.

To verify the effectiveness of the facial expression recognition model proposed in this paper, the recognition accuracy of the NGO-BILSTM face recognition model constructed in this paper is compared with the traditional VGG16 network on the FER2013 dataset. The confusion matrix of face recognition using the two methods is used as the experimental results, and the comparison results are shown in Figure 4.

The average recognition accuracy of this model in the FER2013 dataset is 51.29%, and the recognition accuracy of the VGG16 network is 45.14%. Compared with the VGG16 network, the recognition accuracy of the proposed NGO-BILSTM face recognition model has improved by 6.15%. The facial expression of “happy” is recognized very well, and the accuracy of “fear” is enhanced by 24% compared with the VGG16 network. The accuracy of “disgust” and “sadness” is still very low, although the accuracy of these two expression categories is slightly improved compared with the VGG16 network because the number of images of these two expression categories in the FER2013 dataset is small, and the network cannot This is because the number of pictures of these two categories of facial expressions in the FER2013 dataset is small, and the network is not fully trained for these two categories of facial expressions, so the accuracy of these two categories is relatively low.

This also shows that the model proposed in this paper has a high recognition accuracy and verifies the reliability of the proposed model.

#### 3.2.2. FERPlus dataset

**Figure 5:**
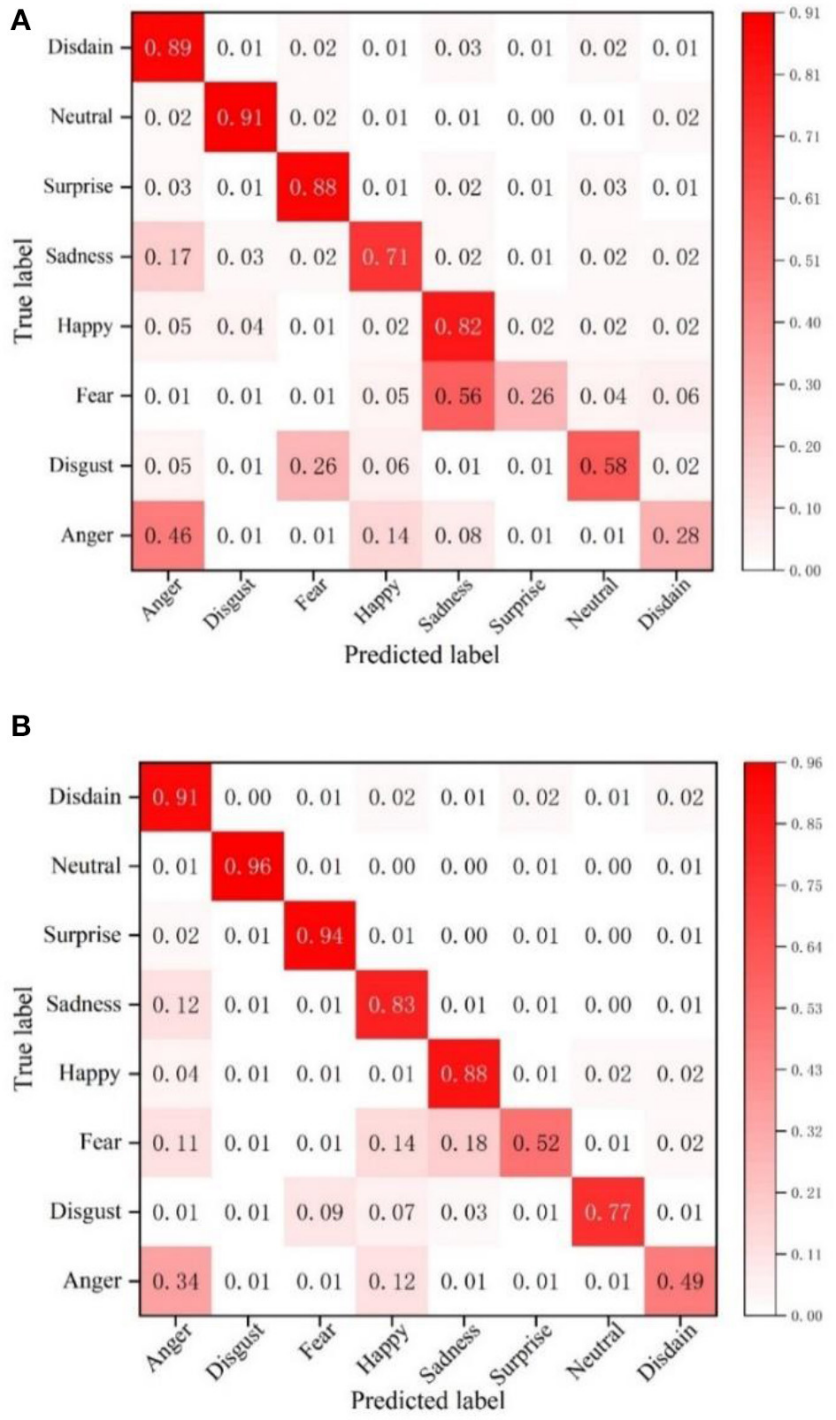
Confusion matrix on the FERPlus dataset. **(A)** Confusion matrix of VGG16 network. **(B)** The confusion matrix of the model in this paper.

To further verify the effectiveness of the method proposed in this chapter, the recognition accuracy of the NGO-BILSTM face recognition model constructed in this paper is compared with that of the traditional VGG16 network on the FERPlus dataset. The confusion matrix of face recognition using the two methods is used as the experimental results, and the comparison results are shown in Figure 5.

The average recognition accuracy of this model on the FERPlus dataset is 78.75%, and the recognition accuracy of the VGG16 network is 66.63%. Compared with the VGG16 network, the recognition accuracy of the NGO-BILSTM face recognition model proposed in this paper has improved by 12.12%. The accuracy of all the eight expression categories is improved, and the accuracy of “neutral” face expression recognition is the highest, reaching 96%, while the accuracy of “fear” face expression recognition is the highest, increasing by 26%. This shows that the proposed NGO-BILSTM-based face expression recognition model has better face expression recognition results on the FERPlus dataset.

#### 3.2.3. RAF-DB dataset

To further verify the effectiveness of the method proposed in this chapter, the recognition accuracy of the NGO-BILSTM face recognition model constructed in this paper is compared with that of DLP-CNN, GACNN, PACNN, and LDL-ALSG on the RAF-DB dataset, and the comparison results are shown in Table 7.

**Table 7:** Accuracy of different models on the RAF-DB dataset.

**Network model**	**Accuracy rate (%)**
DLP-CNN	84.27
GACNN	80.09
PACNN	82.36
LDL-ALSG	86.54
NGO-BILSTM	89.72

The recognition accuracy of the NGO-BILSTM face expression recognition model proposed in this paper is 89.72% on the RAF-DB dataset, which is 5.45, 9.63, 7.36, and 3.18% higher than those of the four methods DLP-CNN, gACNN, pACNN, and LDL-ALSG on the RAF-DB dataset, respectively. This indicates that the facial expression recognition model based on NGO-BILSTM in this paper has higher recognition accuracy and verifies the reliability of the proposed model in this paper.

## 4. Conclusion

To explore the application of the NGO-BILSTM model in facial expression recognition, this paper constructs the NGO-BILSTM face expression recognition model based on the NGO algorithm and BILSTM neural network and uses the loss function with Adam optimizer for weight update. For the effectiveness of the model in this paper, the three face expression datasets of FER2013, FERPlus and RAF-DB are evaluated by the accuracy of the confusion matrix, and the experimental results are as follows:

(1) The average recognition accuracy of this paper's model on the FER2013 dataset is 51.29%, and the recognition accuracy of the VGG16 network is 45.14%. Compared with the VGG16 network, the recognition accuracy of the model proposed in this paper is improved by 6.15%.
(2) The average recognition accuracy of the NGO-BILSTM model proposed in this paper on the FERPlus dataset is 78.75%, and the recognition accuracy of the VGG16 network is 66.63%. Compared with the VGG16 network, the recognition accuracy of the proposed model in this paper is improved by 12.12%.
(3) The identification accuracy of the NGO-BILSTM model proposed in this paper is 89.72% on the RAF-DB dataset, which is 5.45, 9.63, 7.36, and 3.18% higher than the recognition accuracy of the four methods DLP-CNN, gACNN, pACNN, and LDL-ALSG on the RAF-DB dataset, respectively.

This shows that the NGO-BILSTM-based facial expression recognition model proposed in this paper has high recognition accuracy and can be effectively used in facial expression recognition applications.

## Data availability statement

The raw data supporting the conclusions of this article will be made available by the authors, without undue reservation.

## Author contributions

JZ and TC contributed to conception and design of the study. JZ and LY organized the database, performed the statistical analysis, and wrote sections of the manuscript. JZ wrote the first draft of the manuscript. All authors contributed to manuscript revision, read, and approved the submitted version.
